# Molecular characterization of cytolethal distending toxin gene-positive *Escherichia coli* from healthy cattle and swine in Nara, Japan

**DOI:** 10.1186/1471-2180-14-97

**Published:** 2014-04-18

**Authors:** Atsushi Hinenoya, Kensuke Shima, Masahiro Asakura, Kazuhiko Nishimura, Teizo Tsukamoto, Tadasuke Ooka, Tetsuya Hayashi, Thandavarayan Ramamurthy, Shah M Faruque, Shinji Yamasaki

**Affiliations:** 1 Graduate School of Life and Environmental Sciences, Osaka Prefecture University, 1-58 Rinku ourai-kita, Izumisano, Osaka 598-8531, Japan; 2 Division of Microbiology, Department of Infectious Diseases, Faculty of Medicine, University of Miyazaki, 5200 Kiyotake, Miyazaki 889-1692, Japan; 3 National Institute of Cholera and Enteric Diseases, Kolkata 700010, India; 4 Centre for Food and Water Borne Diseases, International Centre for Diarrhoeal Diseases Research, Bangladesh, Dhaka 1212, Bangladesh; 5 Current address: Institute of Medical Microbiology and Hygiene, University of Lübeck, Ratzeburger Allee 160, 23538 Lübeck, Germany

**Keywords:** *Escherichia coli*, Cytolethal distending toxin, *Escherichia albertii*, Molecular typing

## Abstract

**Background:**

Cytolethal distending toxin (CDT)-producing *Escherichia coli* (CTEC) has been isolated from patients with gastrointestinal or urinary tract infection, and sepsis. However, the source of human infection remains unknown. In this study, we attempted to detect and isolate CTEC strains from fecal specimens of healthy farm animals and characterized them phenotypically and genotypically.

**Results:**

By PCR analysis, the *cdtB* gene was detected in 90 and 14 out of 102 and 45 stool specimens of healthy cattle and swine, respectively, and none from 45 chicken samples. Subtypes of the *cdtB* genes (I to V) were further examined by restriction fragment length polymorphism analysis of the amplicons and by type-specific PCRs for the *cdt-III* and *cdt-V* genes. Of the 90 *cdtB* gene-positive cattle samples, 2 *cdt-I*, 25 *cdt-III*, 1 *cdt-IV*, 52 *cdt-V* and 1 both *cdt-III* and *cdt-V* gene-positive strains were isolated while 1 *cdt-II* and 6 *cdt-V* gene-positive were isolated from 14 *cdtB* positive swine samples. Serotypes of some isolates were identical to those of human isolates. Interestingly, a *cdt-II* gene-positive strain isolated from swine was for the first time identified as *Escherichia albertii*. Phylogenetic analysis grouped 87 *E. coli* strains into 77 phylogroup B1, 6 B2, and 4 D, respectively. Most of the B1 strains harbored both *lpfA*_*O113*_ and *ehaA*. Three and twenty-two *cdt-V* gene-positive strains harbored *eaeA* and *stx* genes, respectively, and seven possessed *cdt-V, stx* and *subAB* genes. The *cnf2* gene, normally present in *cdt-III* gene-positive strains, was also detected in *cdt-V* gene-positive strains.

**Conclusions:**

Our results suggest that healthy cattle and swine could be the reservoir of CTEC, and they could be a potential source of human infections.

## Background

Cytolethal distending toxin (CDT) was discovered in an *Escherichia coli* strain isolated from diarrheal patient in 1987 [1]. Since then, expression of CDT has been reported from a variety of pathogenic Gram-negative bacteria, including *Aggregatibacter* (formerly *Actinobacillus*) *actinomycetemcomitans*, *Campylobacter* spp., *Escherichia albertii*, *Haemophilus ducreyi*, *Helicobacter* spp., *Providencia alcalifaciens*, and *Shigella* spp. [2-4].

The *cdt* operon contains three adjacent genes, *cdtA*, *cdtB* and *cdtC*, and expression of all the genes is necessary for maximum toxin activity. While CdtB acts as an active subunit with DNase I activity, CdtA and CdtC facilitate binding of CDT to a yet-to-be-identified receptor molecule(s) on susceptible cells and entry of CdtB into the cytoplasm. As a result, CDT induces distention and eventual death of certain cultured eukaryotic cell lines by causing an irreversible arrest of the cell cycle at the G_1_ or G_2_ phase [4].

In CDT-producing *E. coli* (CTEC), five subtypes of CDT (I through V) have been reported based on the amino acid sequences and the genomic location of their genes [4]. Although CTEC strains have been isolated from children with diarrhea [4], case control studies conducted in children up to 5 years of age in Brazil (used DNA probes for CDT-I) [5], Bangladesh (for CDT-I) [6] and Nigeria (for CDT-I and CDT-II) [7] failed to demonstrate significant association of CTEC with acute diarrhea. However, animal experiments with recombinant CDT of *Shigella dysenteriae* and *Campylobacter jejuni* CDT knockout mutants indicated that CDT is involved in diarrhea and inflammatory response [2]. Moreover, Pandey et al. [8] reported that high titer CDT-I-producing enteropathogenic *E. coli* (EPEC) were isolated from patients with bloody diarrhea in India while low titer producers were isolated from patients with acute watery diarrhea. We also demonstrated that an *E. coli* strain isolated from a child with bloody diarrhea in Japan, which was initially suspected to be Shiga toxin-producing *E. coli* (STEC), did not possess the *stx* genes rather it produced CDT-I by a retrospective analysis [9]. Furthermore, we have recently reported presence of various subtypes of the *cdtB* (*cdt-I* to *cdt-V*) genes in diarrheal stool specimens of children at a high rate (~9.7%). Moreover, out of 30 CTEC isolates, which produced any of the 5 subtypes of CDT (CDT-I to CDT-V), 23 were isolated as a sole pathogen [10] suggesting possible association of CTEC with diarrhea in children.

*E. coli* normally resides in the intestine of warm-blooded animals which are suspected to be the reservoir and possible source of human infection of pathogenic *E. coli*. For example, major natural reservoirs for STEC, one of the most important groups of food-borne pathogens, have been established to be domestic ruminants, such as cattle, sheep, and goats [11]. During the processing of carcasses, fecal contamination or transfer of bacteria from animal’s skin to the carcass can facilitate transmission of STEC to the meat [12]. Indeed, on a number of occasions, CTEC also have been isolated from various farm animals [13-16], and these were associated with diseased animal.

In this study, we attempted to detect *cdtB* gene in stool specimens of apparently healthy domestic animals including cattle, swine and chickens from Nara prefecture in Japan. We further isolated and characterized CTEC strains from these farm animals by serotyping, phylogenetic grouping and virulence gene profiling and compared with the strains of human origin.

## Results

### Detection and isolation of *cdtB* gene-positive bacteria

For analyzing the presence of CTEC in healthy farm animals, 102 stool specimens collected from cattle in a farm and 45 rectal swabs collected from swine and chickens in another farm were subjected to PCR-RFLP analysis which can specifically amplify so far known *E. coli cdtB* genes followed by subtyping them as *cdt-I* to *cdt-V* based on restriction site polymorphism. As shown in Table 1, 90 and 14 samples from cattle and swine, respectively, produced a 588-bp long PCR fragment containing the *cdtB* gene, while no PCR product was obtained using samples of chicken origin. The 90 *cdtB* gene-positive amplicons obtained from cattle stools were found to be comprised of 2 *cdt-I*, 87 *cdt-III*/*V* and 1 *cdt-IV*. Although same number of bacterial strains carrying the *cdt-I* and *cdt-IV* genes was successfully recovered, in the case of *cdt-III/V*, 78 bacterial isolates were obtained out of 87 PCR-positive cases. Similarly, the 14 amplicons derived from swine samples were identified as 1 *cdt-II* and 13 *cdt-III*/*V*. Analysis of bacterial cells allowed us to recover 1 and 6, as *cdt-II* and *cdt-III/V*, respectively (Table 1). The *cdtB*-positive isolates were confirmed to carry *cdtA, cdtB* and *cdtC* genes by colony hybridization using corresponding gene probes (data not shown).

**Table 1:** **Detection of various subtypes of *Escherichia coli cdtB *** **gene in domestic animals by PCR-RFLP**

**Animal**	**No. of samples**	**No. of *cdt *** **positive (%)**	**No. of isolates**	***cdt *** **subtype (PCR/isolate)**			
				***cdt-I***	***cdt-II***	***cdt-III/V***	***cdt-IV***
Cattle	102	90 (88%)	81	2/2	0	87/78	1/1
Swine	45	14 (31%)	7	0	1/1	13/6	0
Chicken	45	0 (0%)	-	-	-	-	-

### Discrimination of *cdt-III/V*-positive bacteria

**Figure 1:**
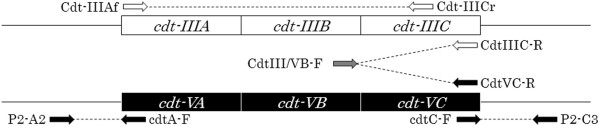
**Schematic representation of PCR primer binding region of type specific PCR for *cdt-III *and *cdt-V*.** White (Cdt-IIIAf, Cdt-IIICr and CdtIIIC-R), black (CdtVC-R, P2-A2, cdtA-F, cdtC-F and P2-C3) and gray (CdtIII/VB-F) arrows indicate PCR primers which specifically bind to *cdt-III*, *cdt-V* and both *cdt-III* and *cdt-V* genes, respectively.

We attempted to further discriminate *cdt-III*/*V*-positive bacteria by type-specific PCR assays as reported previously [10,17]. However, the type-specific PCR failed to differentiate *cdt-III* and *cdt-V* genes in 2 *cdt-V* gene-positive *E. coli* (CTEC-V) OUT:H48, 1 both *cdt-III* and *cdt-V* gene-positive *E. coli* (CTEC-III and V) of cattle, and 5 CTEC-V O98:H10 and 1 OUT:HUT of swine as indicated by asterisk in Table 2. Therefore we developed new type-specific PCR primers for *cdt-III* and *cdt-V* genes in this study as shown in Figure 1. Using these primers all *cdt-III/V* positive isolates were clearly differentiated according to the subtypes of *cdt*, except for one isolate in which both *cdt-III* and *cdt-V* genes were detected as given in Table 2. Finally, among 81 *cdtB* gene-positive isolates of cattle origin, 2 were found to harbor *cdt-I*, 25 *cdt-III*, 1 *cdt-IV*, 52 *cdt-V* and 1 both *cdt-III* and *cdt-V*, whereas 1 and 6 out of 7 *cdtB* gene-positive isolates from swine contained *cdt-II* and *cdt-V*, respectively.

**Table 2:** **Bacteriological characterization, virulence gene profile and *cdt *** **subtype of CDT-producing *Escherichia coli *** **isolated from cattle and swine in Japan**

**Host**	**CDT subtype**	**Serotype**	**PG**^**1**^	**n=**	**CDT-III and -V subtyping**				**Virulence gene**									
									**DEC**^**8**^					**Adhesin**^**9**^				**NTEC**^**10**^
					***cdt*-*IIIi***^**2**^	***cdt-Vi***^**3**^	***cdt-IIIABC***^**4**^	***cdt-V*** **up**^**5**^**/down**^**6**^	***stx1***	***stx2***	***subAB***	***eaeA***	***astA***	***saa***	***lpfAO113***	***ehaA***	***iha***	***cnf2***
Cattle	CDT-I	O112ac:H20	B1	1	ND^7^	ND	ND	ND/ND	-	-	-	-	-	-	+	+	-	-
		OUT:H26	D	1	ND	ND	ND	ND/ND	-	-	-	-	-	-	-	+	-	-
	CDT-IV	O169:H10	B2	1	ND	ND	ND	ND/ND	-	-	-	-	+	-	-	-	-	-
	CDT-III	O2:HUT	B2	3	+	-	+	-/-	-	-	-	-	-	-	-	-	-	+
		O2:NM	B2	1	+	-	+	-/-	-	-	-	-	-	-	-	-	-	+
		O7:H6	B1	1	+	-	+	-/-	-	-	-	-	-	-	+	+	-	+
		O88:H2	B1	1	+	-	+	-/-	-	-	-	-	-	-	+	+	-	+
		O88:H4	B1	1	+	-	+	-/-	-	-	-	-	+	-	+	+	-	+
		O88:H6	B1	1	+	-	+	-/-	-	-	-	-	-	-	+	+	-	+
		OUT:H1	B1	1	+	-	+	-/-	-	-	-	-	-	-	+	+	-	+
		OUT:H21	B1	11	+	-	+	-/-	-	-	-	-	+	-	+	+	-	+
		OUT:H45	D	1	+	-	+	-/-	-	-	-	-	-	-	-	+	-	+
		OUT:HUT	B1	1	+	-	+	-/-	-	-	-	-	-	-	+	+	-	+
		OUT:NM	B1	3	+	-	+	-/-	-	-	-	-	-	-	+	+	-	+
	CDT-V	O2:H10	B2	1	-	+	-	+/+	-	-	-	-	-	-	-	-	-	-
		O8:HUT	B1	1	-	+	-	+/+	-	-	-	-	-	-	+	-	-	+
		O22:H8	B1	5	-	+	-	+/+	+ (4^12^/5^13^)	+ (4/5)	-	-	+ (1/5)	+	+	+	+	-
		O22:HUT	B1	2	-	+	-	+/+	+	+	-	-	-	+	+	+	+	-
		O113:H21	B1	3	-	+	-	+/+	-	+	+	-	-	+	+	+	+	-
		O113:NM	B1	2	-	+	-	+/+	-	+	+	-	-	+	+	+	+	-
		O118:NM	B1	1	-	+	-	+/+	-	-	-	-	+	-	+	+	-	-
		O154:H34	B1	1	-	+	-	+/+	-	-	-	-	-	-	+	+	+	-
		O156:HUT	B1	3	-	+	-	+/+	-	-	-	+^11^	-	-	+	+	-	-
		O163:HUT	B1	1	-	+	-	+/+	-	-	-	-	+	-	+	+	-	-
		OUT:H1	B1	1	-	+	-	+/+	-	-	-	-	-	-	+	-	-	+
		OUT:H19	B1	2	-	+	-	+/+	-	-	-	-	+	-	+	+	-	-
		OUT:H2	B1	5	-	+	-	+/+	-	-	-	-	-	-	+	-	-	+
		OUT:H21	B1	1	-	+	-	+/+	-	-	-	-	-	-	+	+	-	-
		OUT:H25	B1	1	-	+	-	+/+	+	+	+	-	-	+	+	+	+	-
		OUT:H48*	D	2	-	+	-	-/-	-	-	-	-	+	-	-	+	-	-
		OUT:H6	B1	2	-	+	-	+/+	+	+	-	-	-	+	+	+	+	-
		OUT:H8	B1	5	-	+	-	+/+	+	+	-	-	-	+	+	+	+	-
		OUT:HUT	B1	7	-	+	-	+/+	+ (2/7)	+ (2/7)	-	-	+ (4/7)	+ (2/7)	+	+	+ (2/7)	-
		OUT:NM	B1	6	-	+	-	+/+	-	+ (1/6)	+ (1/6)	-	+ (5/6)	+ (1/6)	+	+	+ (1/6)	-
	CDT-III and V	O2:HUT*	B2	1	+	+	-	-/-	-	-	-	-	+	-	-	-	-	-
Swine	CDT-V	O98:H10*	B1	5	-	+	-	-/+	-	-	-	-	+	-	+	+	-	-
		OUT:HUT*	B1	1	-	+	-	-/+	-	-	-	-	+	-	+	+	-	-
	CDT-II	O84:NM^14^	D	1	ND	ND	ND	ND/ND	-	-	-	+	-	-	-	-	-	-

*bfp*, *EAF*, *elt*, *est*, *aggR*, *invE* genes for DEC, *cnf1* for NTEC, and *efa1* for adhesin were negative in all strains tested.

*Not properly differentiated by previous type-specific PCR assays, ^1^phylogenetic group, ^2^PCR result by CdtIII/VB-F and CdtIIIC-R primers, ^3^PCR result by CdtIII/VB-F and CdtVC-R primers, ^4^PCR result by Cdt-IIIAf and Cdt-IIIACr primers ^5^PCR result by P2-A2 and cdtA-F primers, ^6^PCR result by cdtC-F and P2-C3 primers, ^7^not done, ^8^genes for DEC, ^9^genes for Adhesin, ^10^gene for NTEC, ^11^eae-θ/γ2, ^12^No. of positive strains, ^13^No. of tested strains, ^14^identified as *Escherichia albertii*.

### Identification of CTEC

**Figure 2:**
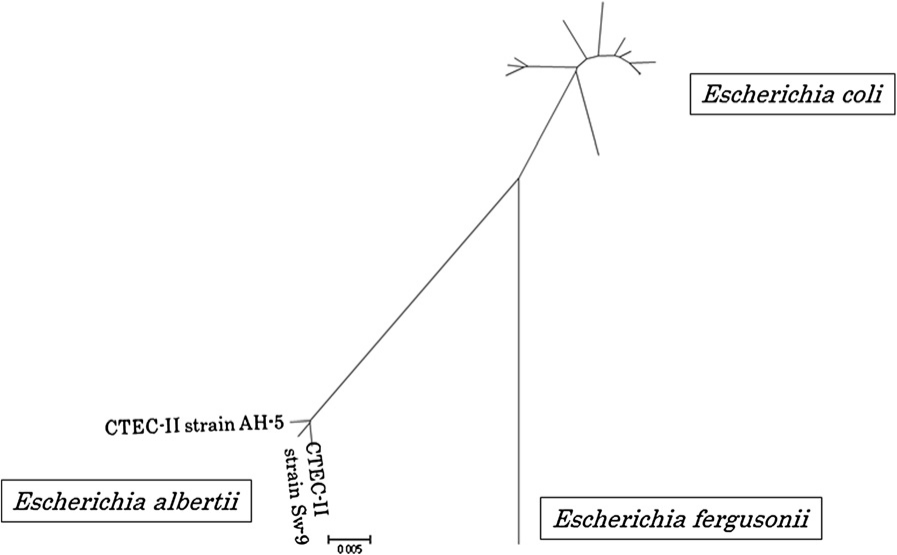
**Neighbor-joining tree based on nucleotide variation at 7 conserved housekeeping loci.** The partial gene sequences for 7 housekeeping loci of CTEC-II strains Sw-9 and AH-5, *E. coli*, *E. fergusonii* and *E. albertii* were concatenated in the order *adk*, *fumC*, *gyrB*, *icd*, *mdh*, *purA* and *recA* and aligned. Based on 3,423 bp of the concatenated sequences, a neighbor-joining tree was constructed by using MEGA 4 software.

All *cdtB* gene-positive isolates from cattle and swine were confirmed as *E. coli* by biochemical tests except for a *cdt-II* gene-positive strain from swine (strain Sw-9). By API 20E testing, the strain Sw-9 was identified as *E. coli* (74.6%) with a doubtful api profile of 51445021 (https://apiweb.biomerieux.com/jsp). However, unlike typical *E. coli*, strain Sw-9 was nonmotile at 37°C and indole-negative, did not ferment lactose and sucrose, and did not produce β-glucuronidase. Partial 16S rRNA gene sequence of strain Sw-9 was identical (452/452 bp; 100%) to that of *E. albertii* (GenBank: HM194884), but also highly similar to those of *Shigella boydii* (GenBank: AY696682; 451/452 bp [99.8%]) and *E. coli* (GenBank: GU237022; 450/452 bp [99.6%]). Sugar utilization tests of dulcitol, D-mannitol, D-melibiose, L-rhamnose and D-xylose also suggested that strain Sw-9 was *E. albertii* and not as *E. coli*[18,19]. Multilocus sequence (MLS) analysis based on the nucleotide sequence variation at 7 housekeeping loci (a total of 3,423 bp) in the genome revealed that strain Sw-9 belongs to the *E. albertii* lineage (Figure 2), consistent with the data of biochemical tests and 16S rRNA gene sequencing. Considering these findings together, the strain Sw-9 was identified as *E. albertii*.

### Serotyping and phylogenetic grouping

To characterize the CTEC strains further, their serotype and phylogenetic groups were determined (Table 2). The 81 cattle isolates were grouped into 12 different O serogroups and 31 O:H serotypes. Two *cdt-I* gene-positive *E. coli* (CTEC-I) isolates were identified as O112ac:H20 (phylogenetic group B1) and OUT:H26 (D), respectively. Three *cdt-III* gene-positive *E. coli* (CTEC-III) isolates were identified as O2:HUT (B2), 16 as OUT (B1) and 1 OUT (D), whereas one each of the 5 CTEC-III isolates belonged to serotype O2:NM (B2), O7:H6 (B1), O88:H2 (B1), O88:H4 (B1), and O88:H6 (B1), respectively. One *cdt-IV* gene-positive *E. coli* (CTEC-IV) isolate was identified as O169:H10 (B2). The CTEC-V isolates belonged to divergent serotypes and phylogenetic groups, including O2:H10 (B2), O8:HUT (B1), O22:H8 (B1), O22:HUT (B1), O113:H21 (B1), O113:NM (B1), O118:NM (B1), O154:H34 (B1), O156:HUT (B1), O163:HUT (B1) and OUT (30 B1 and 2 D strains), as shown in Table 2. One isolate which was positive for both *cdt-III* and *cdt-V* genes was identified as O2:HUT (B2). Five and one CTEC-V isolates from swine were identified as O98:H10 (B1) and OUT:HUT (B1), respectively. Interestingly, the *E. albertii* strain Sw-9 showed cross reaction with the *E. coli* O84 antiserum.

### Virulence gene profile

To analyze the virulence gene profile of the CTEC and *E. albertii* strains isolated in this study, genes for DEC, NTEC and putative adhesins reported in STEC (see details in Material and Methods section) were investigated by colony hybridization assays (Table 2). In agreement with the previous report [20], all the CTEC-III strains possessed the *cnf2* gene, indicating that *cdt-III* of these strains could be located on pVir-like plasmid. Surprisingly, 7 of the CTEC-V strains also possessed *cnf2*.

**Figure 3:**
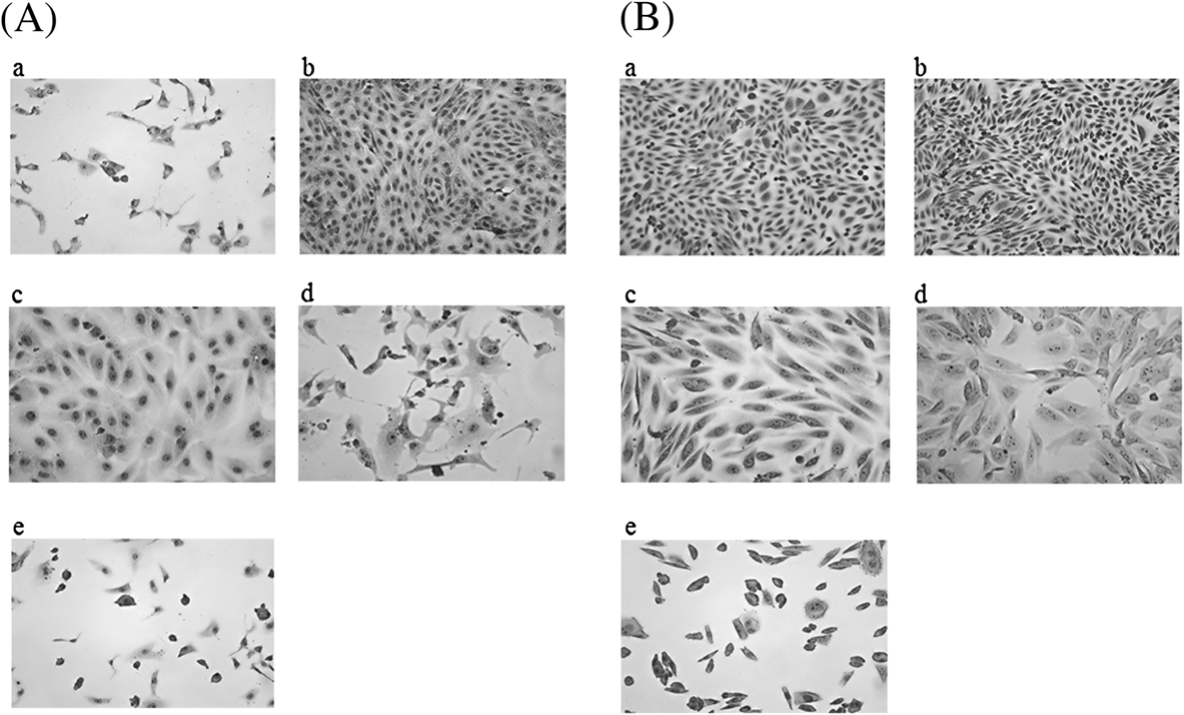
**Cytotoxic effect of sonic lysate of *stx *gene-positive CTEC strains on Vero (A) and CHO cells (B).** Vero and CHO cells were incubated with sonic lysate of *stx* gene-positive CTEC strains for 72 h. The cells were then fixed and observed under microscope (magnification, 200x). STEC strain Sakai (a) and CTEC-I strain GB1371 (c) were used as positive controls for Stx and CDT, respectively. *E. coli* strain C600 (b) was used as negative control. The representative cytotoxicity patterns by CTEC strains positive for *stx*, *cdt-V* (d), and for *stx*, *cdt-V*, *subAB* (e) analyzed in this study are shown.

The *eaeA* gene that encodes an outer membrane protein called intimin, which is necessary for intimate attachment of EPEC and EHEC strains to epithelial cells, was detected in the *E. albertii* strain Sw-9 from swine and all of the 3 CTEC-V O156:HUT (B1) strains from cattle (Table 2). The intimin subtype of three CTEC-V O156 strains was determined as θ/γ2 by PCR-RFLP, but the amplicon was not obtained in *E. albertii* strain Sw-9. Sixteen CTEC-V isolates (6 O22, 10 OUT) were positive for the *stx1* and *stx2* genes, while 6 CTEC-V strains (5 O113, 1 OUT) were positive for only *stx2*. Cytotoxicity assay using Vero and CHO cells, which are susceptible and unsusceptible to Stx intoxication, respectively, indicated that all the *stx* gene-positive CTEC strains produced functional Stx (titer ranging from 16 to 128<) and CDT (1 to 64) (Figure 3). However, 7 strains caused unexpected morphological change to CHO cells, indicating that these strains might produce a third toxin. Since the observed morphological change resembled to that induced by SubAB, an AB_5_ toxin discovered in LEE-negative STEC [21], the 7 strains were subjected to PCR analysis specific to the *subA* and *subB* genes and all the strains were positive for both the genes. Collectively, these data indicate that the 7 *E. coli* strains produced CDT-V, Stx and SubAB toxins.

*stx* gene-positive CTEC strains harbored the putative adhesin genes of STEC such as *saa*, *lpfA*_*O113*_, *ehaA* and *iha*, among which *lpfA*_*O113*_ and *ehaA* may be linked with long-term persistence in cattle [22], Taguchi et al. unpublished]. In addition, 20 (80%) and 21 (84%) of the CTEC-III isolates from cattle and 49 (94%) and 44 (85%) of the CTEC-V isolates also harbored the *lpfA*_*O113*_ and *ehaA* genes, respectively (Table 2). All the 6 CTEC-V strains from swine also harbored both of the *lpfA*_*O113*_ and *ehaA* genes.

### Sequencing of the *cdt-III* and *cdt-V* genes

To confirm the *cdt* subtyping, a total of 20 strains were selected and subjected to *cdt*-gene sequencing as shown in Table 3, including 7 *cnf2*-positive CTEC-V strains, 2 strains which were negative in *cdt-V*-specific PCR using P2-A2 and cdtA-F, and cdtC-F and P2-C3 primer sets (Figure 1), CTEC-III and V, a CTEC-V strain from swine, and 9 additional strains randomly selected from bovine CTEC-V strains. Strains Bv-7, Bv-43, Bv-56, Bv-61, Bv-91 and Bv-98 were found to contain the identical (100% nucleotide sequence identity) *cdt-V* genes to those in human clinical strains 9282/01 (GenBank: AY365042), 5249/01 (GenBank: AY365043), and AH-26 (GenBank: AB472870). The *cdt-V* genes in strains Bv-1, Bv-3, Bv-5, Bv-8, Bv-15, Bv-49, Bv-65, Bv-55, Bv-68, Bv-21, Bv-88 and Bv-100 also showed high sequence similarity (>96% identity) to the *cdt-V* genes (GenBank: AY365042). The *cdt-III* genes in the strain Bv-87 were 98.7, 97.6 and 88.9% identical to the *cdt-III* (GenBank: U89305), *cdt-V* (GenBank: AJ508930) and *cdt-II* (GenBank: U04208) genes, respectively, whereas the *cdt-V* genes in the same strain were 98.3, 97.1 and 89.6% identical to *cdt-V*, *cdt-III* and *cdt-II*, respectively. P2 phage-related sequence was found in the flanking sequences of all the *cdt-V* genes examined. The *cdt-III* and *cdt-V* genes in strain Bv-87 were 97.0% identical to each other. Strain Bv-87 may have both *cdt-III* genes located on the pVir-like plasmid encoding CNF2 and *cdt-V* genes located on bacteriophage.

**Table 3:** **Percentage of nucleotide sequence identity of *cdt *** **genes between selected strains and type strains**

**Strain**	**Serotype**	**PG**	***cdt***	***cdtA***	***cdtB***	***cdtC***
***cnf2*-positive CTEC-V**						
Bv-1	OUT:H1	B1	*cdt-V*^1^ (99.8%)/*cdt-III*^2^ (98.0%)	*cdt-VA* (100%)/*cdt-IIIA* (97.3%)	*cdt-IIIB* (100%)/*cdt-VB* (99.9%)	*cdt-VC* (99.3%)/*cdt-IIIC* (96.2%)
Bv-3	O8:HUT	B1				
Bv-5	OUT:H2	B1				
Bv-8	OUT:H2	B1				
Bv-15	OUT:H2	B1				
Bv-49	OUT:H2	B1				
Bv-65	OUT:H2	B1				
**CTEC-V with untypable cdt genes by previous PCRs**						
Bv-55	OUT:H48	D	*cdt-V* (97.1%)*/cdt-III* (95.9%)	*cdt-VA* (96.4%)/*cdt-IIIA* (94.6%)	*cdt-IIIB* (97.0%)/*cdt-VB* (96.9%)	*cdt-VC* (98.4%)/*cdt-IIIC* (96.0%)
Bv-68	OUT:H48	D				
Sw-26	O98:H10	B1	*cdt-V* (95.8%)/*cdt-III* (95.1%)	*SbcdtA*^3^ (94.5%)/*EacdtA*^4^ (94.2%)	*cdt-IIIB* (99.1%)/*cdt-VB* (99.0%)	*cdt-VC* (97.4%)/*cdt-IIIC* (95.1%)
**CTEC-III and V**						
Bv-87 (*cdt-III*)	O2:HUT	B2	*cdt-III* (98.7%)*/cdt-V* (97.6%)	*cdt-IIIA* (97.6%)/*cdt-VA* (95.1%)	*cdt-IIIB* (100%)/*cdt-VB* (99.9%)	*cdt-IIIC* (98.5%)/*cdt-VC* (97.6%)
Bv-87 (*cdt-V*)			*cdt-V* (98.3%)*/cdt-III* (97.1%)	*cdt-VA* (96.5%)/*cdt-IIIA* (94.7%)	*cdt-IIIB* (99.8%)/*cdt-VB* (99.6%)	*cdt-VC* (98.7%)/*cdt-IIIC* (96.3%)
**Randomly selected 9 strains from CTEC-V**						
Bv-7	O22:HUT	B1	*cdt-V* (100%)*/cdt-III* (98.0%)	*cdt-VA* (100%)/*cdt-IIIA* (97.3%)	*cdt-VB* (100%)*/cdt-IIIB* (99.9%)	*cdt-VC* (100%)/*cdt-IIIC* (96.2%)
Bv-43	O154:H34	B1				
Bv-56	O156:HUT	B1				
Bv-61	OUT:H8	B1				
Bv-91	O22:H8	B1				
Bv-98	O22:H8	B1				
Bv-21	O2:H10	B2	*cdt-V* (99.8%)/*cdt-III* (98.1%)	*cdt-VA* (100%)/*cdt-IIIA* (97.3%)	*cdt-IIIB* (99.9%)/*cdt-VB* (99.8%)	*cdt-VC* (99.5%)/*cdt-IIIC* (96.7%)
Bv-88	OUT:H25	B1	*cdt-V* (99.8%)/*cdt-III* (98.0%)	*cdt-VA* (100%)/*cdt-IIIA* (97.3%)	*cdt-IIIB* (100%)/*cdt-VB* (99.9%)	*cdt-VC* (99.3%)/*cdt-IIIC* (96.2%)
Bv-100	OUT:H21	B1	*cdt-V* (99.7%)/*cdt-III* (98.0%)	*cdt-VA* (99.9%)/*cdt-IIIA* (97.2%)	*cdt-IIIB* (99.9%)/*cdt-VB* (99.8%)	*cdt-VC* (99.5%)/*cdt-IIIC* (96.3%)

^1^From *E. coli* strain 9282/01 (AY365042), ^2^from 1404 (U89305), ^3^from *S. boydii* strain K-1 (AY696753), ^4^from *E. albertii* strain 19982 (AY696755).

Although *cdtB* (99.0% nucleotide sequence identity) and *cdtC* (97.4% identity) in the strain Sw-26 were highly homologous to those of CDT-V (GenBank: AY365042), the *cdtA* was most homologous to that of *S. boydii* CDT (94.5% identity, GenBank: AY696753), followed by *E. albertii* CDT (94.2% identity, GenBank: AY696755), CDT-II (93.1%), CDT-V (91.2%, GenBank: U04208) and CDT-III (91.0%). The *cdtA* genes in other CTEC-V strains Sw-27, Sw-33, Sw-43, Sw-44 and Sw-45 were also identical to that of strain Sw-26. These data suggest that the CTEC-V from swine in this study might harbor chimeric *cdt* genes consisting of *Sbcdt-A* or *Eacdt-A*, *cdt-VB* and *cdt-VC*.

## Discussion

Clinical importance of CTEC in humans including intestinal and extra-intestinal infections is not yet fully understood. Several studies, however, showed that on several occasions CTEC strains were isolated from patients with diarrhea, septicemia, or urinary tract infection [4], suggesting that CTEC might be associated with human diseases. To understand the possible reservoir and potential source of CTEC infection, we have screened feces of healthy farm animals (cattle, swine and chicken) for the presence of *E. coli cdtB* gene by a PCR-RFLP assay, which can detect and differentiate 5 subtypes of the *E. coli cdtB* gene [10]. In addition, we isolated CTEC strains from the *cdtB* gene-positive samples and characterized them for serotypes, virulence gene profiles and phylogenetic groups to compare with those of CTEC strains from diarrheal patients. There is a report regarding the isolation of CDT-V-producing *E. coli* O157 from healthy cattle by Tóth et al. [23]. In most of the previous studies, however, CTEC strains were isolated from diseased animals with various symptoms [13-16]. In this study, to avoid any bias, we have isolated CTEC strains from *cdtB*-positive fecal sample of apparently healthy cattle and swine.

A total of 81 and 7 CTEC strains have been isolated from 90 and 14 *cdtB* gene-positive fecal samples of cattle and swine, respectively (Table 1). The 81 strains from cattle samples were grouped into 12 O serogroups and 31 O:H serotypes (Table 2). In our previous work, we showed that CTEC-I belonging to the O2 serogroup and B2 phylogenetic group was most predominant among the CTEC strains isolated from children with diarrhea in Japan [10]. Although 6 CTEC strains belonged to the O2 serogroup and B2 phylogenetic group were isolated in this study, none of them were CDT-I producers (4 CTEC-III, 1 CTEC-V, and 1 CTEC-III and V). This may be because of different geographical background between clinical and animal samples collected. Alternatively although cattle and swine carry a variety of CTEC strains, all the CTEC strains in cattle and swine may not be associated with human diseases. Since all types of CTEC have been isolated from patients with diarrhea, CTEC strains found in cattle and swine in this study might be associated with human diseases in future. Results obtained in this study indicate that further studies on prevalence of CTEC in food animals in several farms and meats are needed.

Tóth et al. [23] reported the isolation of CDT-V-producing *E. coli* O157 from healthy cattle in Hungary. However, all the CTEC strains isolated in the present study did not belong to O157 serogroup. It might be due to difference of the strategies. In their study, they tried to isolate only *E. coli* O157 from healthy cattle samples by using cefixime-tellurite-sorbitol-MacConkey agar and also by following the International Organization for Standardization reference method (ISO 16654) using an O157-specific immunomagnetic beads. On the other hand, we targeted CTEC by using PCR-RFLP for detection of all five subtypes of the *E. coli cdtB* gene. We further characterized only one strain from each *cdtB* gene-positive sample. Thus, we cannot exclude the possibility that CTEC O157 was present in our samples, but we could not isolate CTEC O157.

Presence of the *cdt-I* and *cdt-IV*, and *cdt-III* genes were reported to be strongly associated with that of the *cnf1* and *cnf2* genes, respectively [13,24]. It has also been reported that the *cdt-III* genes were located on a plasmid harboring the *cnf2* gene [20], whereas *cdt-V* was chromosomal and carried by bacteriophage [25], suggesting that detection of the *cnf2* gene could be one of the genetic markers to differentiate *cdt-III* and *cdt-V* gene-positive strains. Indeed, all the 25 strains with *cdt-III* were also positive for *cnf2*. However, 7 out of the 52 *cdt-V* gene-positive strains from cattle also contained *cnf2* and this gene arrangement has not yet been reported. Since homology between *cdt-III* and *cdt-V* genes is very high (*cdtA*, 97.3%; *cdtB*, 99.7%; *cdtC*, 96.5%) [4], it is difficult to differentiate the *cdt-III* and *cdt-V* genes by PCR, suggesting that some of the *cdt-III* and *cdt-V* genes might have been misidentified. In the present study, three PCR primer sets, cdt-IIIABC, cdt-Vup, cdt-Vdown, each targeting the internal region of *cdt-III*[10], the 5′ and 3′ flanking regions of *cdt-V*[17], failed in producing specific amplicon in 1, 9 and 3 strains, respectively, out of the 58 CTEC-V and 1 CTEC-III and V (Table 2). However, the type-specific PCR developed in this study using two primer sets each targeting *cdt-III* or *cdt-V* (Figure 1) could produce specific amplicon either for *cdt-III* or *cdt-V*. The *cdt-III*- and *cdt-V*-specific PCR designed in this study is more reliable to differentiate these genes and to generate more precise epidemiological data. In fact, using the type-specific PCR, we identified a both *cdt-III* and *cdt-V* gene-positive *E. coli* strain. To our knowledge, this is the first report to describe the isolation of CTEC-III and V strain.

Since reservoir for STEC has been identified to be ruminant such as cattle and this study also indicates that reservoir for CTEC could be the same, similar genes for adhesion might be associated with colonization of both STEC and CTEC. In addition to the *eaeA* gene, *saa*, *iha*, *lpfA*_*O113*_ and *ehaA* genes have also been reported to encode putative adhesins in STEC O157 and non-O157 [26-29]. Recently Wu et al. [22] described a probable association of these 4 genes, in particular *lpfA*_*O113*_ and *ehaA* genes, with the long-term STEC shedding from cattle. When virulence gene profiling, in particular, for adhesin were analyzed in this study, 86 and 83% strains from cattle and swine, respectively, were found to be positive for *lpfA*_*O113*_ and *ehaA* genes, while 100% *stx* gene-positive CTEC isolates were all positive for *saa*, *lpfA*_*O113*_, *ehaA* and *iha* genes. Furthermore, almost all of them were positive for *cdt-III* or *cdt-V* whereas 2 strains were positive for *cdt-I* genes. In this study, 97% of *cdt* genes detected in the feces of cattle was *cdt-III* or *cdt-V* whereas only 2 and 1% of *cdt* genes were *cdt-I* and *cdt-IV*, respectively. Clark et al. [13] also reported that the *cdt-III* genotype was more prevalent in animal strains although the majority of *cdt* genotypes isolated from humans was *cdt-I* and *cdt-IV*[10]. Taken together, these data indicate that LpfA_O113_ and EhaA could be associated with adhesion of CTEC in cattle intestine, especially CTEC-III and CTEC-V.

Strain Sw-9 initially identified as CTEC-II O84:NM by biochemical test was re-identified as *E. albertii,* a newly emerging diarrheagenic pathogen [19], by a MLS analysis and sugar utilization tests. This may be the first report showing isolation of *E. albertii* from swine in Japan. Furthermore, this finding prompted us to reinvestigate if previously identified CTEC-II strains were of *E. albertii* or not. Indeed the CTEC-II strain AH-5, previously identified as OUT:NM [10], was found to be *E. albertii* (Figure 2). Ooka et al. [19] recently reported that 26 out of 179 *eaeA* gene-positive *E. coli* strains, isolated from humans, birds and the environment in Japan, were identified as *E. albertii* by MLS analysis and *cdtB* gene of CDT-II/III/V subtypes group was detected by PCR in all the *E. albertii* strains except 1 strain. EPEC isolates, previously identified as *E. coli* O86:K61 and contained the *cdtB* gene, were also identified as *E. albertii*[30]. The *cdt* genes of *E. albertii* strain 19982 (GenBank: AY696755) are highly homologous to the *cdt-II* genes present in *E. coli* strains. These data suggest that *E. albertii* might have been misidentified as not only EPEC but also CTEC-II. Since there is no reliable method to identify *E. albertii* other than MLS analysis to date, the development of simple and reliable identification method of *E. albertii* is required. The *cdt-II* genes could be one of useful genetic markers for this purpose although discrimination of *E. albertii* from true CTEC-II is still necessary.

## Conclusions

We could isolate a number of CTEC strains from cattle and swine, which had diverse variations in serotype and genotype. Some of the CTEC strains possessed virulence genes associated with human diseases and serotype that are frequently detected among human clinical strains. Thus, cattle and swine could be possible reservoirs of CTEC and serve as potential sources of infection to human. To the best of our knowledge, this might be the first report regarding comprehensive surveillance and characterization of CTEC strains isolated from healthy food animals. Because of the limited number of animals and farms examined, further studies are of course needed to verify the probability that these animals are indeed the source of CTEC infection to humans.

## Methods

### Sample collection

In August 2004 in Japan, stool specimens from the rectum of 102 cattle (around 1 year of age), including 95 cross breeding cattle (from Bv-1 to Bv-95) and 7 Holstein cow (Bv-96 to Bv-102), and rectal swabs from 45 cross breeding swine (<6 month-old) and 45 broiler chickens (<1 year-old) were collected in Nara, Japan. The cattle were kept in several barns in a farm, the swine in several pens in a barn, and the chickens in a windowless broiler house. All the animals were healthy and asymptomatic. The samples were transported to the laboratory at ambient temperature and processed within 6 h of collection. Fecal sampling in the present study was approved by Laboratory of Animal Research of Nara Prefectural Livestock Experiment Station and performed according to the Guidelines for Animal Experimentation of Nara Prefectural Livestock Experiment Station.

### Detection of *cdtB* gene by PCR

Aliquot of stool specimens and rectal swabs were inoculated into 3 mL of tryptic soy broth (Nissui Pharmaceutical Co., Tokyo, Japan) for enrichment and incubated overnight at 37°C with shaking. Fifty microliter of the culture was added into 450 μL of TE buffer (10 mM Tris–HCl, 1 mM EDTA [pH 8.0]) and boiled for 10 min. After centrifugation at 12,000 × *g* for 3 min, the supernatant was used as a template for PCR in a thermal cycler (GeneAmp PCR System 9700; Life Technologies, Carlsbad, CA, USA). In the PCR assay, the *cdtB* gene was detected by using the *cdtB* common primer set which can detect all five subtypes of the *E. coli cdtB* gene [10] (Table 4). *E. coli* strain C600 and *E. coli* O86 strain GB1371 (harboring the *cdt-I* genes) were used as negative and positive controls, respectively. To examine the CDT subtypes, a PCR-RFLP assay was employed as reported previously [10]. Briefly, PCR products were digested by either *Eco*RI/*Eco*RV or *Msp*I (Takara Bio Inc., Shiga, Japan) and the digests were analyzed by electrophoresis in 3.0% agarose gels (NuSieve 3:1 Agarose; Takara Bio Inc.). Since differentiation of *cdt-III* from *cdt-V* by PCR-RFLP assay was not successful, type-specific PCRs were performed to further discriminate *cdt-III* from *cdt-V* by using specific primers such as Cdt-IIIAf and Cdt-IIICr [10], P2-A2/cdtAF and cdtC-F/P2-C3 [17] and newly designed primers such as CdtIII/VB-F/CdtIIIC-R and CdtIII/VB-F/CdtVC-R in this study (Figure 1). The primer sequences and PCR conditions are presented in Table 4.

**Table 4:** PCR primers and conditions used in this study

			**PCR conditions**			**Amplicon**	
**Primer**	**Sequence (5′-3′)**	**Target**	**Denaturing**	**Annealing**	**Extension**	**(bp)**	**Reference**
Cdt-Bcomu	TAAATGGAATATACATGTCCG	*cdt-IB?~?VB*	94°C, 30 s	50°C, 30 s	72°C, 60 s	588	[10]
Cdt-Bcomd	TTTCCAGCTACTGCATAATC						
Cdt-IIIAf	GTAGGCATTCTTATTCCA	*cdt-IIIABC*	94°C, 30 s	50°C, 30 s	72°C, 90 s	1,909	[10]
Cdt-IIICr	AGTTTTCTTATCTGTCGG						
CdtIII/VB-F	CAGAAGGACTCAGATGTC						
CdtIIIC-R	TGGTTGTTTGAGGTCAGT	*cdt-IIIBC*	94°C, 30 s	55°C, 30 s	72°C, 60 s	546	this study
CdtVC-R	GCTCTGTGGTACAACTTC	*cdt-VBC*	94°C, 30 s	55°C, 30 s	72°C, 60 s	537	this study
pVir-u	TCATGTGGAATAACTAGC	*cdt-IIIABC*	94°C, 30 s	52°C, 30 s	72°C, 120 s	2,818	this study
pVir-d	GTTCTGAACTTCACCAG						
EaeA-f	AAACAGGTGAAACTGTTGCC	*eaeA*	94°C, 30 s	50°C, 30 s	72°C, 60 s	454	[10]
EaeA-r	CTCTGCAGATTAACCCTCTGC						
BfpA-f	AATGGTGCTTGCGCTTGCTGC	*bfpA*	94°C, 60 s	56°C, 90 s	72°C, 90 s	324	[10]
BfpA-r	GCCGCTTTATCCAACCTGGTA						
EAF-f	CAGGGTAAAAGAAGATGATAA	EAF	94°C, 60 s	60°C, 90 s	72°C, 90 s	397	[10]
EAF-r	TATGGGGACCATGTATTATCA						
Est-f	ATTTTTMTTTCTGTATTRTCTT	*est*	94°C, 30 s	50°C, 30 s	72°C, 60 s	190	[10]
Est-r	CACCCGGTACARGCAGGATT						
Elt-f	GGCGACAGATTATACCGTGC	*elt*	94°C, 30 s	54°C, 30 s	72°C, 60 s	450	[10]
Elt-r	CGGTCTCTATATTCCCTGTT						
AstA-f	CACAGTATATCCGAAGGC	*astA*	94°C, 60 s	53°C, 60 s	72°C, 60 s	94	[10]
AstA-r	CGAGTGACGGCTTTGTAG						
Eagg-f	CTGGCGAAAGACTGTATCAT	*aggR*	94°C, 60 s	53°C, 60 s	72°C, 60 s	630	[10]
Eagg-r	CAATGTATAGAAATCCGCTGTT						
EVT1	CAACACTGGATGATCTCAG	*stx1*	94°C, 30 s	55°C, 30 s	72°C, 60 s	349	[10]
EVT2	CCCCCTCAACTGCTAATA						
EVS1	ATCAGTCGTCACTCACTGGT	*stx2*	94°C, 30 s	55°C, 30 s	72°C, 60 s	110	[10]
EVC2	CTGCTGTCACAGTGACAAA						
CNF1-f	GGGGGAAGTACAGAAGAATTA	*cnf1*	94°C, 60 s	55°C, 60 s	72°C, 60 s	1,112	[10]
CNF1-r	TTGCCGTCCACTCTCACCAGT						
CNF2-f	TATCATACGGCAGGAGGAAGCACC	*cnf2*	94°C, 60 s	55°C, 60 s	72°C, 60 s	1,241	[10]
CNF2-r	GTCACAATAGACAATAATTTTCCG						
InvE-f	AGTTCTCGGATGCTATGCTC	*invE*	94°C, 30 s	60°C, 30 s	72°C, 60 s	293	[10]
InvE-r	CAAGATTTAACCTTCGTCAACC						
Saa-f	ACCTTCATGGCAACGAG	*saa*	94°C, 30 s	57°C, 30 s	72°C, 60 s	1,504	[23]
Saa-r	AATGGACATGCCTGTGG						
Iha-f	GAAATCAGCATCCGAGG	*iha*	94°C, 30 s	55°C, 30 s	72°C, 60 s	410	[23]
Iha-r	ATACGCGTGGCTGCTG						
Efa1-f	GTCAAAGGTGTTACAGAG	*efa1*	94°C, 30 s	55°C, 30 s	72°C, 60 s	640	[23]
Efa1-r	ATTCCATCCATCAGGCC						
LpfAO113-f	ACTTGTGAAGTTACCTCC	*lpfAO113*	94°C, 30 s	55°C, 30 s	72°C, 60 s	360	[23]
LpfAO113-r	CGGTATAAGCAGAGTCG						
EhaA-f	AGGCATGAGACACGATC	*ehaA*	94°C, 30 s	55°C, 30 s	72°C, 60 s	500	[23]
EhaA-r	AAGTCGTGCCATTGAGC						
SubA-f	GTACGGACTAACAGGGAACTG	*subA*	94°C, 30 s	55°C, 30 s	72°C, 60 s	1,264	[22]
SubA-r	ATCGTCATATGCACCTCCG						
SubB-f	GTAGATAAAGTGACAGAAGGG	*subB*	94°C, 30 s	55°C, 30 s	72°C, 60 s	715	[22]
SubB-r	GCAAAAGCCTTCGTGTAGTC						
P2-A2	CACTGACAACGGCTGAAC	Upstream	94°C, 30 s	55°C, 30 s	72°C, 60 s	848	[18]
cdtA-F	AAATGGGGAGCAGGATAC	of *cdt-VA*					
cdtC-F	GAACCCCAAATACAGACC	Downstream	94°C, 30 s	55°C, 30 s	72°C, 60 s	712	[18]
P2-C3	TGGTTGATGACGGTGTTA	of *cdt-VC*					
eae-F	AGGATATTCTTTCTCTGAATA	*eaeA*	94°C, 30 s	55°C, 30 s	72°C, 60 s	1,300	[33]
eae-R	ATATYTATTTGCWGSVCCCCAT						

### Identification of *cdt*-harboring organisms

Enrichment culture in which *cdtB* gene was detected by the PCR was serially diluted in sterile 10 mM phosphate buffered saline (pH 7.4) and 100 μL of each dilution was spread on MacConkey agar (Difco Laboratories, Detroit, MI, USA). Colonies were transferred to nitrocellulose (Schleicher & Schuell, Dassel, Germany) or Hybond-N^+^ membrane (GE Healthcare, Buckinghamshire, UK) by a replica blotting method and a colony hybridization assay was carried out by using specific DNA probes under high stringent condition. For preparation of probes, the *cdt-IB*, *cdt-IIB*, *cdt-IIIB* and *cdt-IVB* genes were PCR amplified using template DNAs isolated from *E. coli* strains NT3363 [8], AH-5, AH-6 and AH-8 [10], respectively, and common primer sets (Table 4) followed by labeling of each PCR product by random priming method using the MultiPrime DNA Labeling System (GE Healthcare) and (α-^32^P)-dCTP (111 TBq/mmol) (Perkin Elmer, Wellesley, MA, USA). Hybridization positive colonies were detected from the corresponding master plate and reconfirmed by *cdtB*-specific PCR using the common primers (Table 4). To identify *cdtB*-positive colonies as *E. coli*, bacterial cells were further analyzed by the API 20E System (bioMérieux, Marcy-l’Etoile, France) and by conventional biochemical tests [31]. When the results of biochemical tests were ambiguous, further confirmation was done by 16S rRNA gene sequencing (approximately 500 bp in size) by using the MicroSeq 500 16S rDNA Sequencing Kit and an ABI PRISM 3100 Genetic Analyzer (Life Technologies). Serotyping was carried out by tube agglutination method using somatic (O1-O173) and flagellar (H1-H56) antisera [31], which were prepared at the Osaka Prefectural Institute of Public Health, Osaka, Japan.

### Multilocus sequence analysis

Multilocus sequence (MLS) analysis was applied to the *cdt-II*-positive strain according to the protocol by University of Warwick (http://mlst.warwick.ac.uk) with minor modifications. Briefly, partial gene sequences for 7 housekeeping loci (*adk*, *fumC*, *gyrB*, *icd*, *mdh*, *purA*, *recA*) were determined by sequencing their PCR products using the BigDye Terminator Sequencing Kit (Life Technologies). Obtained sequences were aligned and trimmed to a uniform size by using Seqman (DNASTAR, Madison, WI, USA) and concatenated. Based on the concatenated sequences, a neighbor-joining tree was constructed using the MEGA 4 software. Following *E. coli*, *E. fergusonii* and *E. albertii* strains were included in the MLS analysis as references: *E. coli* strains K-12 (GenBank: NC000913), ED1a (GenBank: CU928162), HS (GenBank: CP000802), and SE11 (GenBank: AP009240), uropathogenic *E. coli* strains 536 (GenBank: CP000247), and IAI39 (GenBank: CU928164), avian-pathogenic *E. coli* strain O1 (GenBank: CP000468), enteroaggregative *E. coli* (EAEC) strain 55989 (GenBank: CU928145), enterotoxigenic *E. coli* (ETEC) strain E24377A (GenBank: CP000800), STEC O157:H7 strain Sakai (GenBank: BA000007), O26 strain 11368 (GenBank: AP010953), O103 strain 12009 (GenBank: AP010958), CDT-II-producing *E. coli* (CTEC-II) strain AH-5 [10], *E. fergusonii* strain ATCC 35469 (GenBank: CU928158) and *E. albertii* strain LMG20976 [32].

### Phylogenetic grouping of CTEC

Phylogenetic groups of each CTEC isolates were determined by PCR developed by Clermont et al. [33].

### Detection of virulence genes

Presence of virulence genes including *cdt* in diarrheagenic *E. coli* (DEC) and necrotoxigenic *E. coli* (NTEC) and putative adhesin genes of STEC were analyzed by colony hybridization assays using appropriate DNA probes (Table 2) as described previously [10,22]. CTEC strain GB1371 (*cdt-IA*, *cdt-IC*, *eaeA*, *bfpA*, EAF), ETEC strains 12566 (*elt*) and 12671 (*est*), EAEC strain O42 (*aggR*, *astA*), STEC O157:H7 strain Sakai (*stx1*, *stx2*, *iha*, *efa1*, *ehaA*), STEC O113:NM strain D-129 (*subAB*, *saa*, *lpfA*_*O113*_) [Taguchi et al. unpublished], enteroinvasive *E. coli* strain 3 (*invE*), CTEC strains AH-1 (*cnf1*), AH-5 (*cdt-IIA*, *cdt-IIC*), AH-6 (*cdt-IIIA*, *cdt-IIIC*, *cnf2*), AH-8 (*cdt-IVA*, *cdt-IVC*) and AH-10 (*cdt-VA* and *cdt-VC*) were used as positive controls. The DNAs of these control strains were also used as template to PCR amplify each of the virulence gene followed by preparation of DNA probes. The *E. coli eaeA* gene was PCR amplified using the eae-F and eae-R primer set and subtyped by PCR-RFLP with *Msp*I as described previously [34].

### Cytotoxicity assay

Cytotoxicity assay was performed as described earlier [10]. Briefly, test strains were grown overnight in 3 mL of tryptic soy broth at 37°C overnight with shaking. Bacterial cells were lysed by sonication using an Astrason ultrasonic processor (Heat-System 7 Ultrasonics, Farmingdale, NY, USA) and each sonic lysate was passed through sterile disposable filter with 0.22-μm pore size and each filtrate was used for cytotoxicity assay. Vero and CHO cells were seeded at density of 1 × 10^4^ cells in a 96 well plate (Asahi glass Co., Ltd., Tokyo, Japan) respectively, and 20 μL of 2-fold serially diluted each toxin solution was added to assay their cytotoxic effects. After 9 h of incubation, 100 μL of fresh medium was added per well and cytotoxic effect of each test sample, if any, was examined microscopically after 72 h of incubation. The toxin titer was expressed as the reciprocal of the highest dilution that caused 50% of the Vero and CHO cells in a well to be killed and distended, respectively. *E. coli* strains Sakai and GB1371 were always used as positive controls and as a negative control we used *E. coli* strain C600. Vero and CHO cells were cultured in Minimum Essential Medium (MEM) and MEM-α (Life technologies), respectively, containing 10% fetal bovine serum (EuroClone S.p.A., Pero, Italy), and 1% antibiotic-antimycotic (100x) (Penicillin G sodium [10,000 U/mL], streptomycin sulfate [10,000 μg/mL], and 25 μg/mL amphotericin B in 0.85% saline [Life technologies]). Cells were cultured at 37°C under 5% CO_2_ in air.

### Sequence analysis of *cdt-III* and *cdt-V*

To determine the entire sequence of the *cdt* genes, the *cdt* gene-cluster including their flanking regions were PCR amplified followed by sequencing as previously described [10]. For the *cdt-III* genes, PCR product obtained by the pVir-u and pVir-d primers specific to the flanking region of *cdt-III* on the pVir plasmid was sequenced. For the *cdt-V* genes, PCR products obtained by the P2-A2 and CdtVC-D2 primers and the CdtIII/VB-F2 and P2-C3 primers were sequenced (Figure 1). Each PCR product was purified by the QIAquick PCR Purification Kit (QIAGEN, Hilden, Germany) and the nucleotide sequence of the PCR product was determined as described above. Nucleotide and amino acid sequences were analyzed and compared with each subtype using the BLAST program through the DDBJ (DNA Data Bank of Japan), and the DNA Lasergene software package (DNASTAR).

### Nucleotide sequence accession numbers

All nucleotide sequences obtained in this study have been registered in the DDBJ database. The accession numbers are AB839651-AB839676 (for the *cdt* genes) and AB839677-AB839690 (for 7 housekeeping genes used for MLS analysis).

## Competing interests

The authors declare that they have no competing interests.

## Authors’ contributions

Conception and design of the study: AH, MA, KN, SY. Laboratory work: AH, KS, MA, TT. Data analysis and interpretation: AH, TO, TH, TR, SMF, SY. Manuscript writing: AH, TR, SMF, SY. All authors read and approved the final manuscript.
